# Impact of processing parameters on the interfacial bonding and properties of recycled LCS/WC–Co bilayers developed through powder metallurgy

**DOI:** 10.1038/s41598-025-26946-6

**Published:** 2026-03-17

**Authors:** Mostafa M. Abdelhaleem, A. A. El-Daly, Omayma A. Elkady, Mohamed Hassan, Mahmoud Atta

**Affiliations:** 1 Mechanical Design and Production Engineering Department, Zagazig University, Zagazig, 44519 Egypt; 2 Physics Department, Faculty of science, Zagazig University, Zagazig, Egypt; 3 Powder Technology Department, Central Metallurgical R&D Institute (CMRDI), Cairo, Egypt

**Keywords:** Bi-Material, Bonding interface, Diametral compression, PM method, Cemented tungsten carbide, Recycling LCS, Engineering, Materials science

## Abstract

This study aims to develop a tough-hard (LCS/WC-Co) bilayer composite using recycled low-carbon steel (LCS) with WC–Co through conventional powder metallurgy (PM), offering a cost-effective and sustainable route to enhance mechanical performance. Processing parameters like sintering temperature, compaction pressure, and particle size, were optimized to control microstructural development and mechanical behavior. The microstructure results show that the defect-free interfaces, dense layer and strong interfacial bonding strength are achieved at the optimal parameters of 1300 °C, 313 MPa, and 25 μm particle size. Lower sintering temperatures (< 1280 °C) produced porosity and weak adhesion, whereas sintering above 1320 °C led to interfacial cracking. At the interface, mutual diffusion occurred with Fe diffusion into WC–Co and Co migrating into LCS. Concurrently, WC decomposition facilitated the formation of Fe(W) and CoFe intermetallic, together with minor Co₃W₃C and Fe₃W₃C phases. These interfacial reactions provided strong cohesion and enhanced mechanical performance, yielding compressive and tensile interfacial bonding strength of 209 MPa and 44 MPa, with hardness of 150 ± 6 HV for the LCS layer and 660 ± 70 HV for the WC–Co layer.

## Introduction

 The growing demand for multifunctional composite materials is rising, due to their enhanced flexibility compared to single materials, which combining materials with different properties that can significantly improve the performance and reliability in industrial applications^1,2^. Driven by the high production costs and inherent brittleness of cemented carbide, research and industry are tracking steel–cemented carbide (LCS/WC-Co) bilayer composite joints as alternatives to conventional WC–Co^3^. Such composites are engineered to unite the toughness of steel with the outstanding hardness, wear resistance, and thermal resilience of WC–Co carbide^4^. Cemented tungsten carbide (WC–Co) is commonly used in a wide range of applications such as drilling and cutting tools in the machining industries due to its excellent strength, high thermal stability, corrosion, abrasion and wear resistant property^5–7^. These novel systems aims to combine the ductility and toughness of low carbon steel (LCS)^8^ with the high hardness, wear resistance, and thermal resilience of WC-Co, thereby expanding their potential industrial applications.

In recent years, various techniques including welding^9^, tape casting^10^, hot pressing^11^, brazing^12^, and powder metallurgy (PM) have been employed to fabricate multifunctional composite materials. Among them, PM offers a particularly efficient and economical route, as it enables the integration of dissimilar materials while allowing near-net-shape parts^13,14^. The method consists of three fundamental stages: powder mixing, co-compaction into a porous green compact, and co-sintering, which results in dense and mechanically sound component exhibiting the desired functional properties^15^. However, joining metals and ceramics presents significant challenges owing to the substantial mismatch in coefficients of thermal expansion (CTE), melting points, and shrinkage rates between the two materials. While metals shrink by ~ 6% during sintering, ceramics shrink only ~ 1%, leading to interfacial stress and cracks either during sintering or cooling. These effects promote delamination and weaken the integrity of laminated composites such as iron–WC systems^16,17^.

Resent research indicates that controlling sintering temperature is essential for strong metal–ceramic bonds. Boonyongmaneerat and Schuh^18^ found that increased densification initially enhances adhesion in W/Al₂O₃ bilayers, but exceeding a certain density can lead to excessive shrinkage differences and then weakens the inherent strength of their bond. Using powder metallurgy, the optimal cohesion was achieved at 1280 °C following 1080 °C pre-sintering^19^. Gustafson^20^ identified 1180–1330 °C as the suitable range for WC/Fe systems, while Pascal et al.^21^ reported effective steel/cemented carbide bilayer formation at 1280 °C with minimal shrinkage mismatch.

Hence, careful selection and monitoring of the co-sintering temperature are essential to achieve desirable properties and high-quality sintered bilayer compacts. For instance, Thomazic et al.^22^ found that densification in cemented carbide/steel bilayers was governed by carbon content, temperature, and time, whereas pressure and heating rate were insignificant, and full densification was only achieved beyond 1290 °C. Also, Ojo-Kupoluyi et al.^23^ investigated WC–Fe–C/Fe–W–C bilayers prepared by PM, highlighting the effects of carbon content under sintering conditions of 1290–1295 °C. However, the strength and integrity of co-sintered multilayers are strongly influenced by powder composition and sintering conditions, where inadequate control may result in defects such as mismatch, fracture, cracking, delamination, or the formation of unwanted phases^24,25^.

Recycling clean metallic chips also enhances sustainability by reducing raw material use. Recycled low-carbon steel (LCS) was selected as it offers economic and environmental benefits while maintaining metallurgical characteristics comparable to common LCS. This choice avoids the high cost of producing or purchasing steel powders, providing a more sustainable alternative^26–29^. Although submicron to medium-grained WC–Co with plate-like morphologies has been extensively studied, coarse-grained WC–Co and recycled LCS remain underexplored despite their importance for optimizing these materials. These materials require higher sintering temperatures for densification and refinement, yet little is known about how processing conditions influence their microstructure, cohesion, and mechanical performance in bilayer composites.

Therefore, this study aims to fabricates recycled LCS/WC–Co bilayer composites using conventional powder metallurgy and examines their interfacial adhesion under varied processing conditions. The effects of particle size, compaction pressure, and sintering temperature on density, shrinkage mismatch, and bonding strength are systematically evaluated. Microstructural and phase analyses using OM, SEM–EDX and XRD confirm the interface quality, while optimized parameters yield bilayers with enhanced interfacial bonding strength, tailored hardness, and reduced shrinkage mismatch. The work further discusses the mechanisms governing LCS/WC–Co interface formation.

## Materials and methods

### Materials

**Fig. 1:**
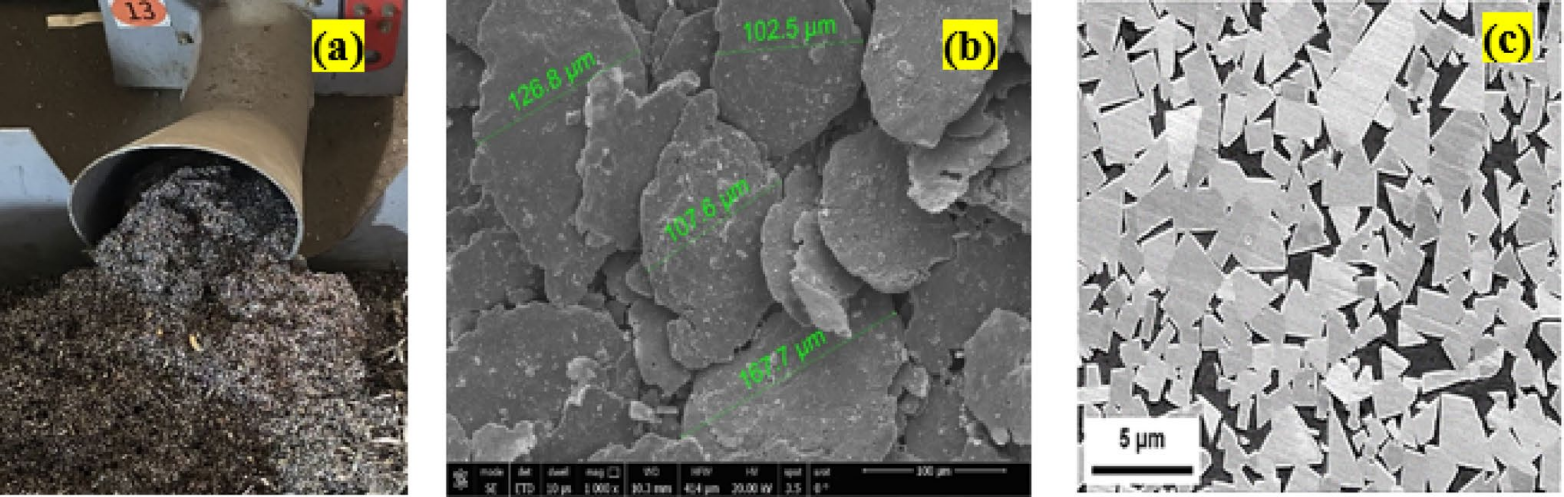
Conversion of CNC-machined waste chips into fine powders for use in the base layer substrates: (**a**) initial waste metal chips, (**b**) SEM micrograph of recycled LCS powder, and (**c**) SEM micrograph ofobtained WC–12%Co powder.

Bi-metallic specimens were produced with a recycled low-carbon steel (LCS) substrate and a WC–12% Co composite overlay. Waste LCS chips obtained from CNC machining were processed into powders (< 125 μm) using the Fullenwider method^30^, consisting of chemical cleaning, crushing, and mechanical ball milling, followed by sieving into three size fractions. WC–12% Co powder (grade YG-12) was supplied by NILCCo., Egypt, and underwent ball milling and sieving. Both LCS and WC powders were milled under an argon atmosphere to avoid oxidation, employing a 5:1 ball-to-powder ratio. Milling durations were 30 h for LCS and 12 h for WC, at 300 rpm. Figure 1 illustrates the original LCS chips and SEM micrographs of the prepared powders, with corresponding chemical compositions given in Table 1.

**Table 1:** Chemical composition of WC-Co and LCS powders (wt%).

Material	C	Al	Si	Mn	W	Co	Ni	Fe
WC-Co	47	-	-	-	40.7	12.3	-	-
LCS	0.2	0.1	0.4	0.6	-	-	0.08	Bal.

**Table 2:** Parameters processing data used in PM process.

Particles size (LCS and WC) (μm)	<25**,** 25 up to 63**,** 63 up to125
Compaction pressure (MPa)	208**,** 260**,** 313
Sintering temperature (^◦^C)	1260**,** 1280**,** 1300**,** 1320**,** 1340

### Specimen fabrication

**Fig. 2:**
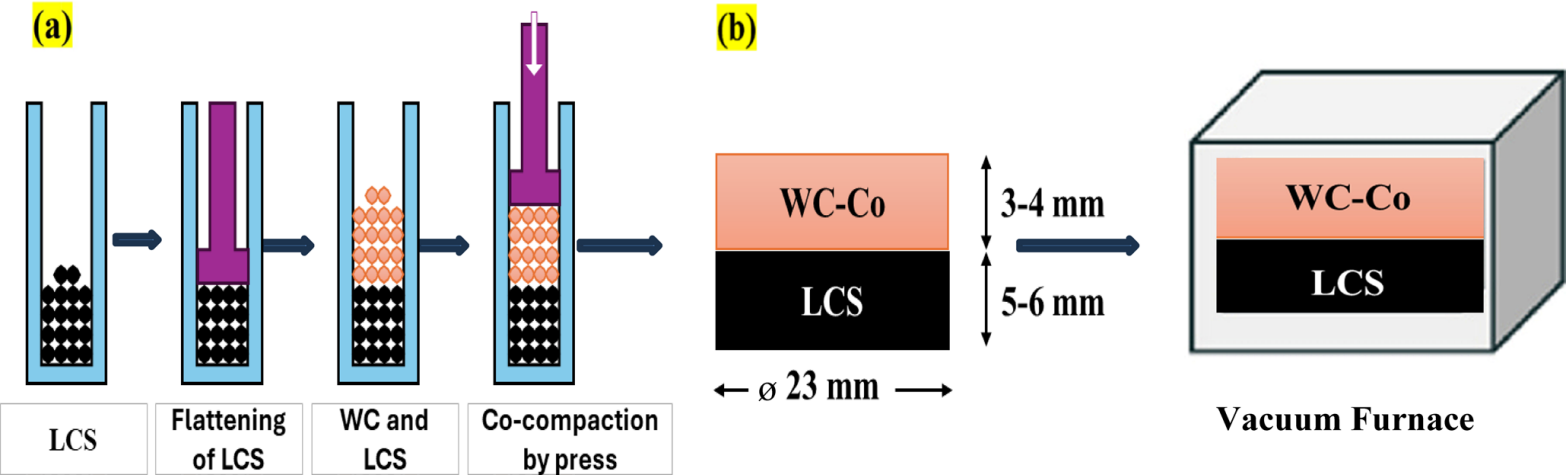
**(a)** Schematic diagram of the co-compaction LCS/WC-Co procedures, and (**b)** Final compacted LCS/WC-Co bilayer specimen and sintering in a vacuum furnace.

Bi-metallic LCS/WC–12%Co materials were fabricated using the conventional powder metallurgy (PM) route, chosen for its cost-effectiveness, simplicity (powder preparation, mixing, compaction, and sintering), and ability to produce near-net-shape components^31^. A 1 wt% paraffin wax binder was incorporated into both powders and heated to 100 °C to enhance compaction. Bilayer green compacts were prepared by hydraulic pressing under pressures of 208, 260, and 313 MPa to optimize both green density and interfacial adhesion. The compaction procedures and the produced bilayer specimens are depicted in Fig. 2, following the method reported in^32^. The compacted bilayer specimens were sintered in a vacuum furnace (MTI XD-1400MT). According to literature, the powder sintering temperature is typically 0.7–0.9 of the melting point of the composite constituents^33^. Considering the difference in sintering ranges between LCS (1100–1300 °C)^34^ and WC–Co (1300–1500 °C)^35^, sintering was conducted between 1260 °C and 1340 °C to investigate bonding quality. The heating cycle included three key stages: (i) paraffin binder removal at 250 °C, (ii) transformation of the LCS from a BCC to FCC phase at 730°C^36^, and (iii) The onset of intermediate solid-state sintering occurs around 1100 °C. At this stage, enhanced diffusion and particle rearrangement, promoting grain growth and densification^37^.

**Fig. 3:**
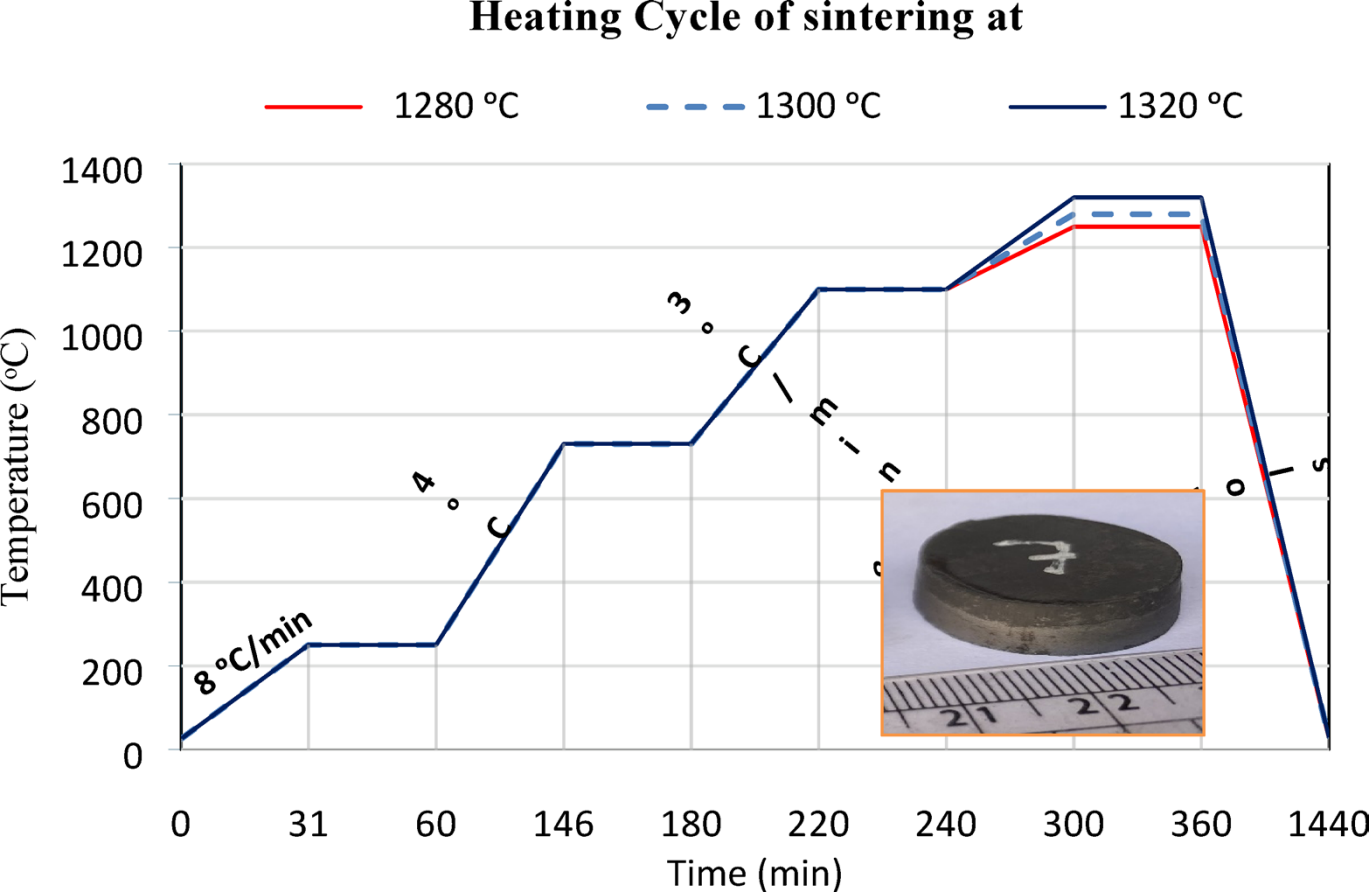
The heating cycle of sintering process for the compacted samples.

Bonding during sintering was governed by a transient liquid phase (TLP) process, wherein molten cobalt reacted with WC to generate a temporary interfacial liquid layer. This layer promoted diffusion and improved bonding with the LCS substrate^38^. After the onset of TLP bonding, the specimens were subjected to isothermal sintering for 1 h, followed by gradual cooling within the furnace. The heating schedule is displayed in Fig. 3.

Accordingly, this study examined three key parameters: particle size, compaction pressure, and sintering temperature. These factors were selected to evaluate their impact on the properties of the bilayer composites, as illustrated in Table 2. To optimize the experimental design, Taguchi’s L15 orthogonal array^39^ was employed, allowing efficient assessment with a limited number of trials. The detailed processing conditions for the fifteen specimens are presented in Table 3.

### Physical measurements and examinations

Relative density, porosity ratio, and shrinkage mismatch are key factors in assessing sintering performance, as they influence the final properties of the samples.

#### Relative density

Specimen density was measured by the Archimedes method (ASTM C-20/87). The true (actual) density was calculated from Eq. (1) and relative density from Eq. (2), indicating sinterability and densification.

1 $$\:\mathrm{Actual}\;\mathrm{density(}{{\rho}}_{\mathrm{a}}\mathrm{)}=\frac{\mathrm{w}\mathrm{e}\mathrm{i}\mathrm{g}\mathrm{h}\mathrm{t}\:\mathrm{i}\mathrm{n}\:\mathrm{a}\mathrm{i}\mathrm{r}}{(\mathrm{w}\mathrm{e}\mathrm{i}\mathrm{g}\mathrm{h}\mathrm{t}\:\mathrm{i}\mathrm{n}\:\mathrm{a}\mathrm{i}\mathrm{r}-\mathrm{w}\mathrm{e}\mathrm{i}\mathrm{g}\mathrm{h}\mathrm{t}\:\mathrm{i}\mathrm{n}\:\mathrm{w}\mathrm{a}\mathrm{t}\mathrm{e}\mathrm{r})}$$

2 $$\:\mathrm{R}\mathrm{e}\mathrm{l}\mathrm{a}\mathrm{t}\mathrm{i}\mathrm{v}\mathrm{e}\:\mathrm{d}\mathrm{e}\mathrm{n}\mathrm{s}\mathrm{i}\mathrm{t}\mathrm{y}\:\left({{\rho}}_{\mathrm{r}}\right)\:=\:{{\rho}}_{\mathrm{a}}/{{\rho}}_{\mathrm{t}\mathrm{h}}$$

where;$$\:{{\rho}}_{\mathrm{t}\mathrm{h}}\:\:\:\mathrm{i}\mathrm{s}\:\mathrm{t}\mathrm{h}\mathrm{e}\:\mathrm{t}\mathrm{h}\mathrm{e}\mathrm{o}\mathrm{r}\mathrm{e}\mathrm{t}\mathrm{i}\mathrm{c}\mathrm{a}\mathrm{l}\:\mathrm{d}\mathrm{e}\mathrm{n}\mathrm{s}\mathrm{i}\mathrm{t}\mathrm{y}\:\mathrm{i}\mathrm{n}\:\mathrm{a}\mathrm{l}\mathrm{l}\:\mathrm{l}\mathrm{a}\mathrm{y}\mathrm{e}\mathrm{r}\mathrm{s},\:$$

$$\:\mathrm{a}\mathrm{n}\mathrm{d}\:\mathrm{c}\mathrm{a}\mathrm{l}\mathrm{c}\mathrm{u}\mathrm{l}\mathrm{a}\mathrm{t}\mathrm{e}\mathrm{d}\:\mathrm{f}\mathrm{r}\mathrm{o}\mathrm{m}\:\mathrm{r}\mathrm{u}\mathrm{l}\mathrm{e}\:\mathrm{o}\mathrm{f}\:\mathrm{m}\mathrm{i}\mathrm{x}\mathrm{t}\mathrm{u}\mathrm{r}\mathrm{e},\:{{\rho}}_{\mathrm{t}\mathrm{h}}=\:{\Sigma\:}\:\left({\rho}\mathrm{i}\:\mathrm{*}\:{\mathrm{M}}_{\mathrm{f}\mathrm{i}}\right)$$ $$\:{{\rho}}_{\mathrm{i}}\:\:\:\:\:\:\mathrm{i}\mathrm{s}\:\mathrm{t}\mathrm{h}\mathrm{e}\mathrm{o}\mathrm{r}\mathrm{e}\mathrm{t}\mathrm{i}\mathrm{c}\mathrm{a}\mathrm{l}\:\mathrm{d}\mathrm{e}\mathrm{n}\mathrm{s}\mathrm{i}\mathrm{t}\mathrm{y}\:\mathrm{f}\mathrm{o}\mathrm{r}\:\mathrm{o}\mathrm{n}\mathrm{e}\:\mathrm{l}\mathrm{a}\mathrm{y}\mathrm{e}\mathrm{r}\:,\:{{\rho}}_{\mathrm{i}}=\frac{1}{{\sum}\:\left[\:\frac{\mathrm{W}\mathrm{i}\:{\%}}{{{\rho}}_{\mathrm{e}}\text{}\mathrm{i}}\:\right]}$$

$$\:{\mathrm{M}}_{\mathrm{f}\mathrm{i}}\:\:\:\mathrm{i}\mathrm{s}\:\mathrm{t}\mathrm{h}\mathrm{e}\:\mathrm{m}\mathrm{a}\mathrm{s}\mathrm{s}\:\mathrm{f}\mathrm{r}\mathrm{a}\mathrm{c}\mathrm{t}\mathrm{i}\mathrm{o}\mathrm{n}\:\mathrm{o}\mathrm{f}\:\mathrm{e}\mathrm{a}\mathrm{c}\mathrm{h}\:\mathrm{l}\mathrm{a}\mathrm{y}\mathrm{e}\mathrm{r}\:,\:{\mathrm{M}}_{\mathrm{f}\mathrm{i}}=\frac{{\mathrm{m}}_{\mathrm{i}}\:\:}{{\mathrm{m}}_{\mathrm{t}\mathrm{o}\mathrm{t}}}$$

$$\:{\mathrm{m}}_{\mathrm{i}}\:\:\:\:\mathrm{i}\mathrm{s}\:\mathrm{m}\mathrm{a}\mathrm{s}\mathrm{s}\:\mathrm{o}\mathrm{f}\:\mathrm{o}\mathrm{n}\mathrm{e}\:\mathrm{l}\mathrm{a}\mathrm{y}\mathrm{e}\mathrm{r}\:\:\:\:\:$$and, $$\:{\mathrm{m}}_{\mathrm{t}\mathrm{o}\mathrm{t}}\:\:\:\:\mathrm{i}\mathrm{s}\:\mathrm{t}\mathrm{o}\mathrm{t}\mathrm{a}\mathrm{l}\:\mathrm{m}\mathrm{a}\mathrm{s}\mathrm{s}\:\mathrm{o}\mathrm{f}\:\mathrm{c}\mathrm{o}\mathrm{m}\mathrm{p}\mathrm{o}\mathrm{s}\mathrm{i}\mathrm{t}\mathrm{e}.$$

#### Porosity percentage (voids content)

The porosity ratio, reflecting sintering quality and densification, is obtained from Eq. (3)

3 $$\:\mathrm{P}\mathrm{o}\mathrm{r}\mathrm{o}\mathrm{s}\mathrm{i}\mathrm{t}\mathrm{y}\:\left(\mathrm{P}\right)\:=\frac{(\:{\mathrm{W}}_{\mathrm{s}}-{\mathrm{W}}_{\mathrm{d}}\:)\:}{({\mathrm{W}}_{\mathrm{s}}-{\mathrm{W}}_{\mathrm{i}}\:)}\times\:\:100$$

where; $\:{\mathrm{W}}_{\mathrm{s}}\:\:$is weight of sample in air (gm), $\:{\mathrm{W}}_{\mathrm{d}}$ is weight of dry sample (gm), and $\:{\mathrm{W}}_{\mathrm{i}}$ is weight of sample in water (gm).

#### Shrinkage volume mismatch

Sintering causes dimensional shrinkage due to particle rearrangement and densification, influenced by powder nature, size, and shape. Height (H) and diameter (D) were measured before and after sintering using a digital Vernier caliper (0.02 mm accuracy). Shrinkage anisotropy (A), from Eq. (4), reflects directional variation: A = 0 indicates uniform shrinkage; positive means radial > axial; negative means axial > radial. Radial Shrinkage Difference (RSD %), calculated from Eqs. (5–6), compares steel and carbide layers. A high RSD signals strong mismatch, leading to interfacial stress and possible defects like cracks or delamination.

4 $$\:\mathrm{S}\mathrm{h}\mathrm{r}\mathrm{i}\mathrm{n}\mathrm{k}\mathrm{a}\mathrm{g}\mathrm{e}\:\mathrm{a}\mathrm{n}\mathrm{i}\mathrm{s}\mathrm{o}\mathrm{t}\mathrm{r}\mathrm{o}\mathrm{p}\mathrm{y}\:\left(\mathrm{A}\right)=\frac{({\Delta\:}\mathrm{D}/\mathrm{D}_0)\:}{({\Delta\:}\mathrm{H}/\mathrm{H}_0)}-1$$

5 $$\:\mathrm{R}\mathrm{a}\mathrm{d}\mathrm{i}\mathrm{a}\mathrm{l}\:\mathrm{S}\mathrm{h}\mathrm{r}\mathrm{i}\mathrm{n}\mathrm{k}\mathrm{a}\mathrm{g}\mathrm{e}\:\mathrm{D}\mathrm{i}\mathrm{f}\mathrm{f}\mathrm{e}\mathrm{r}\mathrm{e}\mathrm{n}\mathrm{c}\mathrm{e}\:\left(\mathrm{R}\mathrm{S}\mathrm{D}{\%}\right)=\left({RS}_{layer\left(WC\right)}-{RS}_{layer\left(LCS\right)}\right)\times\:100$$

6 $$\:\mathrm{R}\mathrm{a}\mathrm{d}\mathrm{i}\mathrm{a}\mathrm{l}\:\mathrm{s}\mathrm{h}\mathrm{r}\mathrm{i}\mathrm{n}\mathrm{k}\mathrm{a}\mathrm{g}\mathrm{e}\:\left({RS}_{layer}\right)=\frac{{\mathrm{D}}_{f}-\:{\mathrm{D}}_{0}}{{\mathrm{D}}_{0}}\times\:100$$

Where, (ΔD/D_o_) is relative shrinkage in the radial direction, and (ΔH/H_o_**)** is relative shrinkage in the height (axial) direction.

#### Microstructure examinations

Bilayer interfaces were examined after preparing specimens via EDM cutting, grinding, polishing, and Murakami etching (10 wt% KOH + K₃Fe(CN)₆)^32^. The microstructures of bilayers and diffusion-bonded joints were examined by Optical Microscope (BX41M-LED) with software OLYMPUS Stream Essentials 2.1. Scanning electron microscope (SEM, JEDL- JSM 5400LV) equipped with an energy-dispersive X-ray spectrometry (EDX) and XRD examination is applied to obtain the intermetallic compounds (IMCs) and chemical composition.

### Mechanical properties characterizations

#### Hardness test

Hardness was measured using Vickers tester (OMPTA/HV-30) per ASTM E384. A diamond pyramid indenter (136°) was applied with a 10 kgf load for 15 s. Indentation diagonals (d_1_, d_2_) were measured, with five tests per bilayer surface averaged. The hardness value was determined using Eq. (7).

7 $$\:HV=\frac{2F\:sin\:\frac{136^\circ\:}{2}}{{d}^{2}}\:$$

Where, F = Load in kg_f_, d = Arithmetic means of the two diagonals, d_1_ and d_2_ in mm. Which can be simplified into the equation: $\:HV=\frac{1.85\:F\:}{{d}^{2}}$

#### Diametrical compressive test

**Fig. 4:**
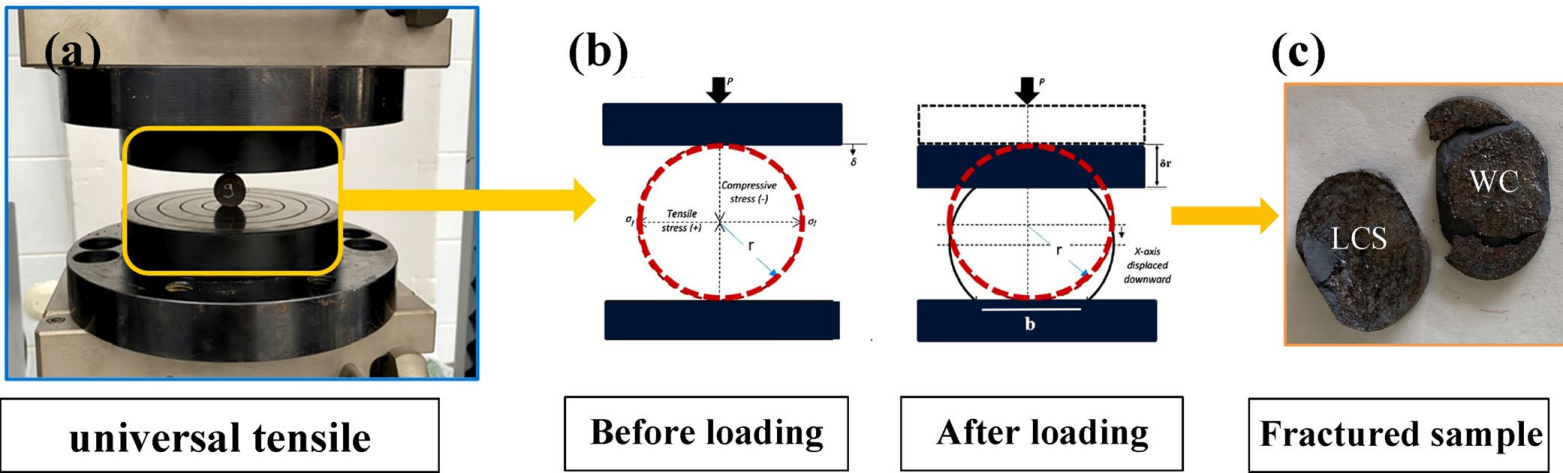
**(a)** Enlargement of fixed specimen in universal tensile machine, **(b)** a schematic diagram of diametrical compression testing method (Brazilian test) before and after loading, and **(c)** the bilayer fractured into two halves upon completion of the test.

The diametrical test examined LCS/WC–Co bilayer adhesion and fracture mechanics via load–displacement analysis^40–42^. Tests were run on a Universal Ibertest Machine (TESTCOM-100) per ASTM D3967-08, t/D = 0.3, crosshead speed 0.1 mm/min (Fig. 4a). Compressive (σ_c_) and tensile (σ_t_) strengths were obtained using Eqs. (8, 9) using the stress analysis framework described in^43,44^ and illustrated in Fig. 4(b). The tensile strength came from the failure load/circumference ratio, while the compressive strength was determined using the same applied load perpendicular to the flattened surface, with width (b) obtained from Eq. (10), assuming that the flattened thickness remained constant^45^.

8 $$\:{\sigma\:}_{t}=\frac{\mathrm{P}}{{\uppi\:}\:\left(\mathrm{r}\times\:\mathrm{t}\right)}$$

9 $${\sigma\:}_{c}=\frac{\mathrm{P}}{\left(\mathrm{b}\times\:\mathrm{t}\right)}\:$$

10 $$\:b=2\times\:\sqrt{{r}^{2}-{\left(r-0.5\delta\:r\right)}^{2}}\:\:$$

where, $\:\mathrm{P}$ is the applied load at bonding failure, $\:\mathrm{t}$ is the thickness of the disk, $\:\mathrm{r}$ is the radius of disk, and $\:\delta\:r$ is measured radial deformation.

## Results and discussions

**Fig. 5:**
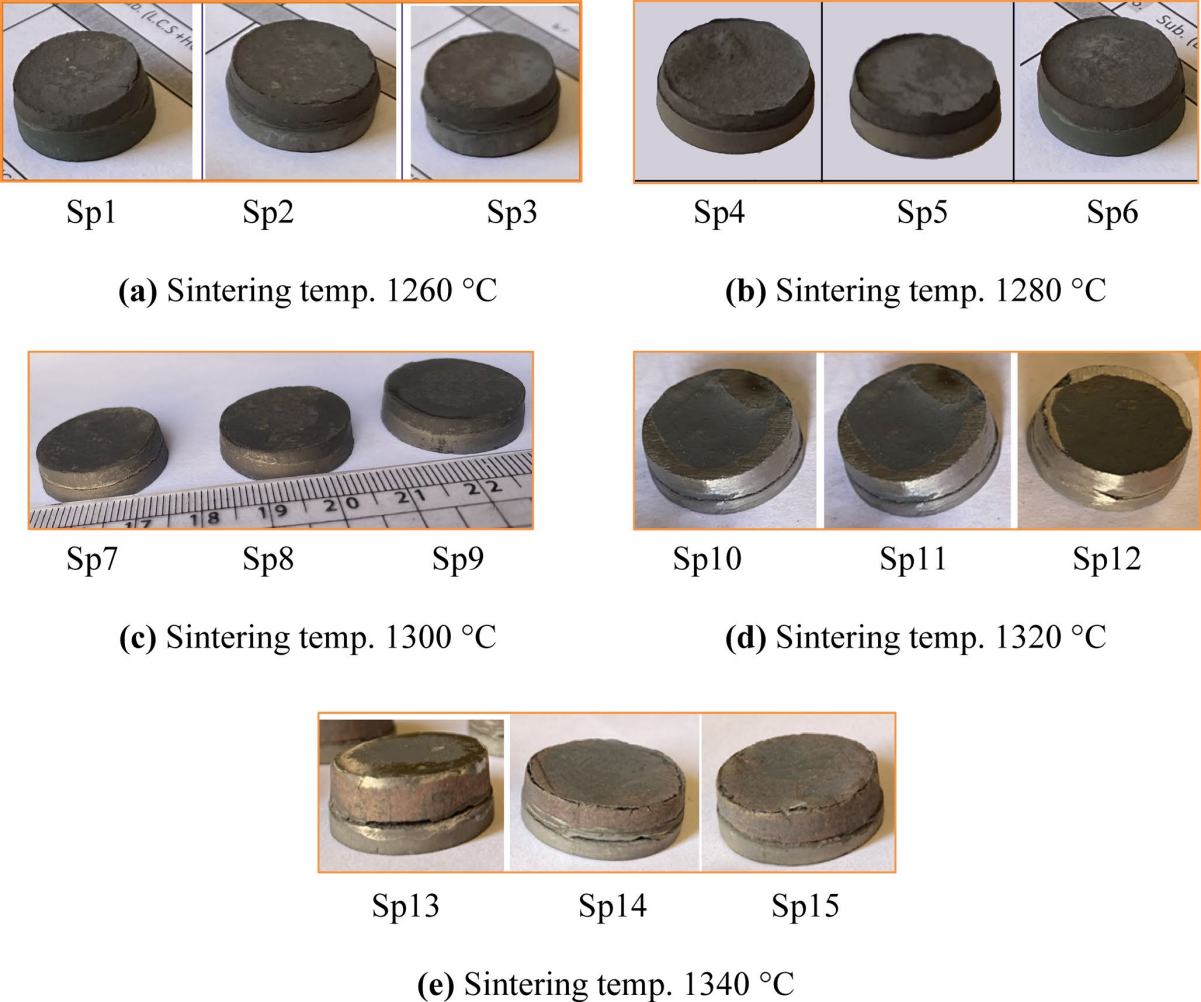
Sintered bilayered samples at different manufacturing parameters, as described in Table 3.

Fifteen (LCS/WC-12%Co) bilayer disk specimens were fabricated by PM based on Taguchi design, with variations in particle size, compaction pressure and sintering temperature. By visual inspection, specimens exhibiting complete sintering and strong interfacial bonding were selected for further testing. The sintered samples, arranged in order of increasing temperature consistent with Table 3, are shown in Fig. 5.

At 1260 °C (Fig. 5a), the WC-Co overlays showed cracks and incomplete densification, indicating that the sintering temperature was insufficient. It is important to note that the peak sintering temperature of 1260 °C remained below the melting point of cobalt. As Petersson^46^ reported, the solidus and liquidus of WC-12%Co are 1285 °C and 1327 °C, respectively, meaning that the reaction becomes thermodynamically favorable only at higher temperatures.

**Fig. 6:**
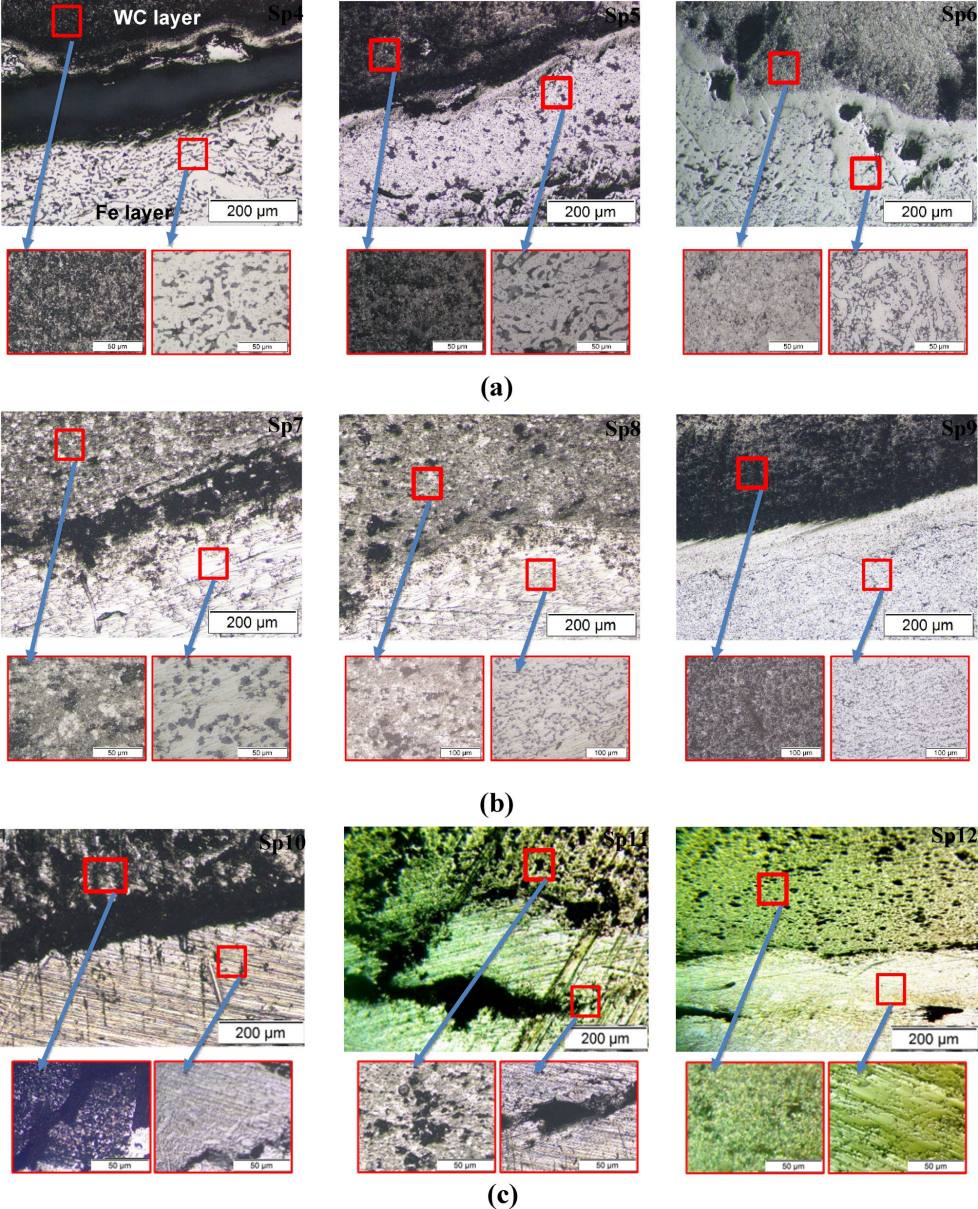
Effect of sintering temperature, particle size and applied pressure on: **(a)** The relative density, **(b)** Shrinkage mismatch.

In contrast, at 1340 °C the interface between the WC-12%Co layer and the LCS substrate showed a visible gap with no metallurgical bonding, as illustrated in Fig. 5(e). Excessive heat likely caused partial melting of LCS, volumetric swelling and layer separation, which hindered neck growth and diffusion, promoting crack formation^21^. Additionally, differential shrinkage from densification mismatch and thermal expansion generated tensile stresses during cooling, further intensifying interfacial cracking^14^.

Most specimens exhibited excellent bonding, particularly those sintered in the 1280–1320 °C range, as shown in Fig. 5(b–d). In this range, the layers compacted into a single cohesive unit due to Fe diffusion into WC and Co diffusion into LCS, forming a strong metallurgical bond which is further confirmed by SEM and EDX analyses. In contrast, specimens sintered at 1260 °C and 1340 °C [Fig. 5(a, e)] were excluded. Therefore, only specimens Sp4–Sp12 [Fig. 5(b–d)] were selected for full characterization of density, shrinkage, hardness, and diametrical compression tests.

### Optical microscopic investigation

Figure 6 presents optical micrographs of the LCS/WC–Co bilayer samples, with focus on the interface region under varying sintering conditions. In Fig. 6(a), specimens Sp4–Sp6 (sintered at 1280 °C) exhibit interfacial voids due to insufficient WC–Co densification and limited material diffusion; the largest gap appears in Sp4 and diminishes through Sp5 to Sp6 correlating with decreasing the WC particle size and increasing the compacting pressure. In Fig. 6(b), samples Sp7–Sp9 (sintered at 1300 °C) show markedly improved bonding: only Sp7 fabricated with the largest particles (125 μm) and lowest pressure (208 MPa) retains a visible gap, while pore prevalence diminishes in Sp8 and Sp9 as the particle size decreases and pressure increases. This enhancement reflects the optimal sintering temperature’s promotion of neck growth at the interface. Conversely, samples Sp10–Sp12 sintered at 1320 °C reveal some interfacial cracking, which intensifies with the larger particle sizes and lower pressures (Fig. 6c). These cracks arise from two principal factors: (i) increased differential shrinkage and residual stresses due to the mismatch in coefficients of thermal expansion between Fe and WC, which inhibit stress relaxation during cooling^18,47^; and (ii) partial melting of impurities in the recycled iron, leading to layer separation Overall, the highest interfacial adhesion was achieved in Sp9 (25 μm particles, 313 MPa, 1300 °C), where the combination of fine WC grains and high compaction promoted extensive diffusion, strong interlocking, and effective surface contact, resulting in maximum bond integrity at the interface^48^.

**Fig. 7:**
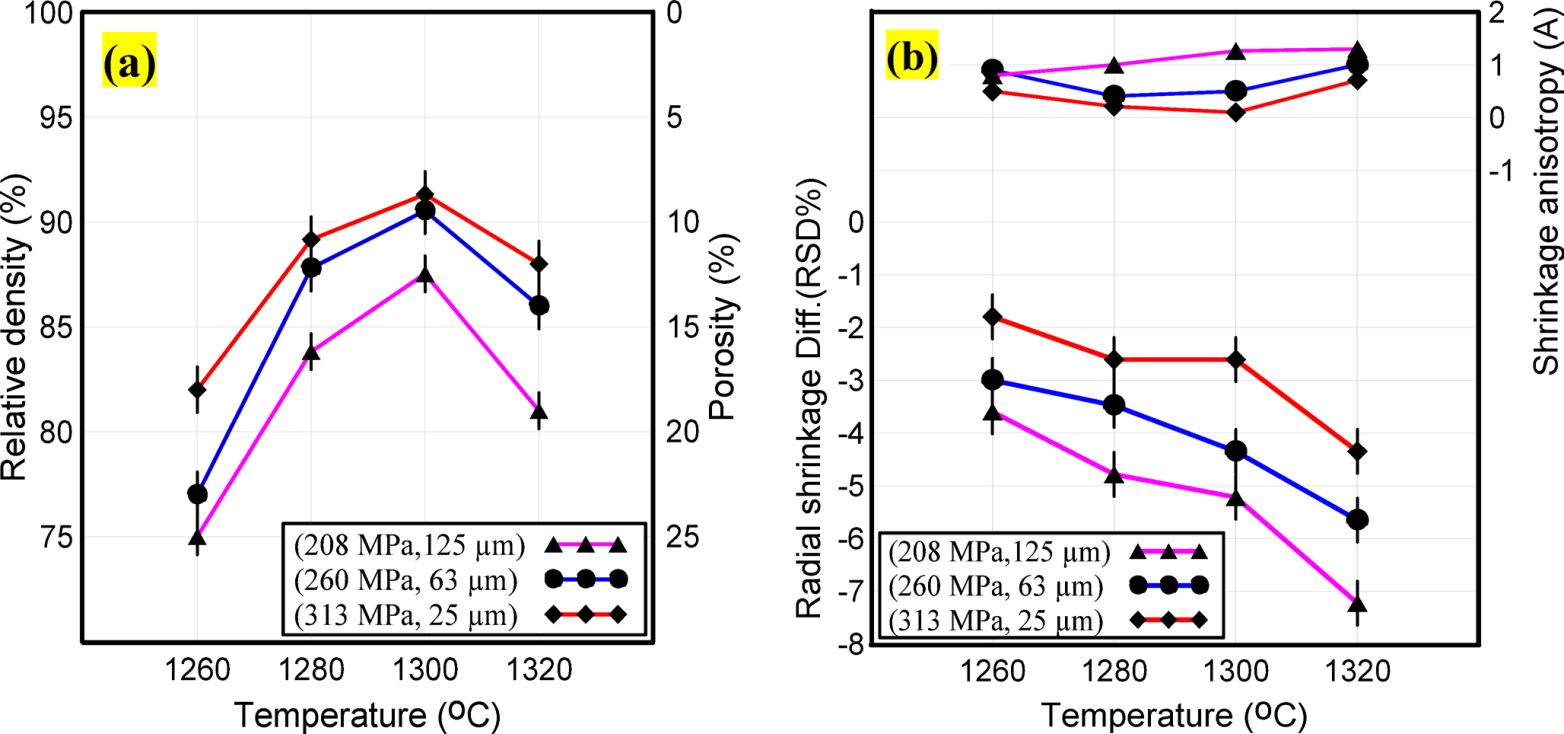
Optical images of the interface between two layers for samples at **(a)** 1280 ᵒC, **(b)** 1300 ᵒC and **(c)** 1320 ᵒC sintering temperatures.

### Relative density and porosity

Figure 7(a) illustrates the influence of processing parameters on the relative density of LCS/WC–Co bilayers. Two main trends are evident. First, relative density increases as the sintering temperature rises from 1260 °C to 1300 °C. Below 1280 °C, the Co binder remains largely solid, restricting particle rearrangement and pore elimination. At 1300 °C, partial melting of Co enhances neck growth and diffusion, producing maximum densities (~ 88% at 208 MPa, ~ 90% at 260 MPa, and ~ 92% at 313 MPa). This effect is mirrored by the inverse porosity axis, which shows minimal porosity (~ 8% at 1300 °C, 313 MPa/25 µm). Nonetheless, full densification of WC–Co is not reached, in agreement with Su^49^, who noted that coarse-grained WC–Co composites require higher sintering temperatures. Second, an increase in sintering temperature to 1320 °C causes a reduction in relative density across all specimens. This decline is linked to thermal stresses generated by mismatched coefficients of thermal expansion (CTE) between the layers, producing swelling, interfacial delamination, and microcracking. Optical microscopy (Fig. 6c) confirms pore re-opening, which lowers bulk density. In contrast, higher compaction pressure and finer LCS/WC grain sizes consistently enhanced densification at all temperatures. Finer powders provided greater surface area for diffusion, while elevated green density promoted pore elimination. Thus, 1300 °C, combined with fine particles and high compaction pressure, is identified as the optimal condition for achieving sufficient densification and robust interfacial bonding in LCS/WC–Co bilayers.

### Shrinkage mismatch

Figure 7(b) shows the influence of sintering temperature, particle size, and applied pressure on the shrinkage of the sintered bilayer samples. It was observed that smaller particle sizes initiate the reduced shrinkage, especially at the lower sintering temperature of 1260 °C. The differential shrinkage at temperature of 1280 °C is almost like that of sintered at 1300 °C; however, the higher temperature (1300 °C) improved interfacial cohesion without significant liquid phase infiltration, on account of reducing shrinkage mismatch of bilayers. As the temperature increases, the radial shrinkage difference (RSD) also increases. This behavior is primarily attributed to the mismatch in CTE between LCS and the WC–Co layer. LCS exhibits greater thermal expansion under heat due to its lower thermal stability, whereas WC–Co remains more dimensionally stable^17^. Furthermore, the calculated shrinkage anisotropy (A) at the value near to zero indicates that samples sintered at 1300 °C exhibit the most compatible and uniform shrinkage of two layers in the radial and axial directions.

We deduce that the optimized processing parameters (25 μm particle size, 313 MPa compaction pressure, and sintering at 1300 °C) effectively reduced shrinkage mismatch, resulting in a dense bilayer with improved interfacial bonding between the two layers.

### XRD examination

**Fig. 8:**
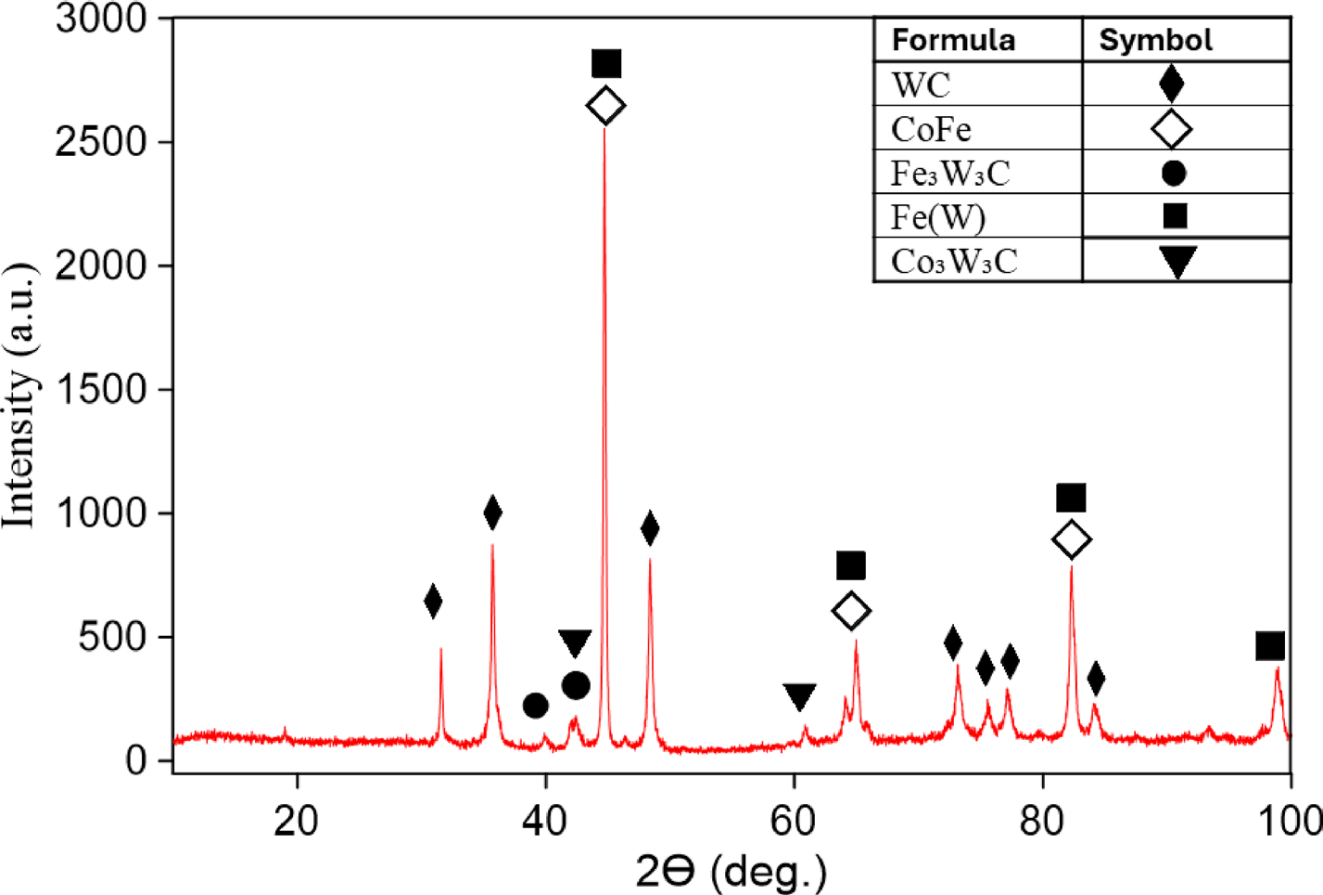
XRD spectrum of the bilayered (LCS/WC-Co) for Sp. 9 sintered at 1300 °C.

Figure 8 shows the XRD analysis of the bilayer material of sample 9, which is considered the best, in terms of density and interfacial bonding. The result reveals significant phase transformations resulting from interfacial diffusion during the sintering process. The dominant phase identified is iron tungsten Fe(W)^50^, indicating extensive diffusion between the Fe-based layer and tungsten carbide, generally described as part of the Fe–W system known to form through interdiffusion and phase interactions between the two layers^18–21^. Additionally, a substantial amount of CoFe intermetallic compound (IMC) was detected, suggesting strong interactions between the cobalt binder and iron matrix. Notably, the original WC phase confirms that a significant portion of tungsten carbide was consumed in the formation of new phases. Minor phases such as iron tungsten carbide (Fe₃W₃C)^51^ and cobalt tungsten carbide (Co₃W₃C)^51^, commonly referred to as η-phases, further support the occurrence of complex solid-state reactions at the interface. Although such transformations can improve interfacial adhesion, they inevitably bring about a considerable loss of hardness, as the intrinsically hard WC phase is replaced by comparatively softer intermetallic phases. This observation is consistent with the measured decrease in the hardness of WC layer and the corresponding increase in hardness of LCS substrate in the bilayer specimens. The findings underscore the inherent trade-off between interfacial bonding strength and overall mechanical performance in such composite systems.

### SEM and EDX scan

**Fig. 9:**
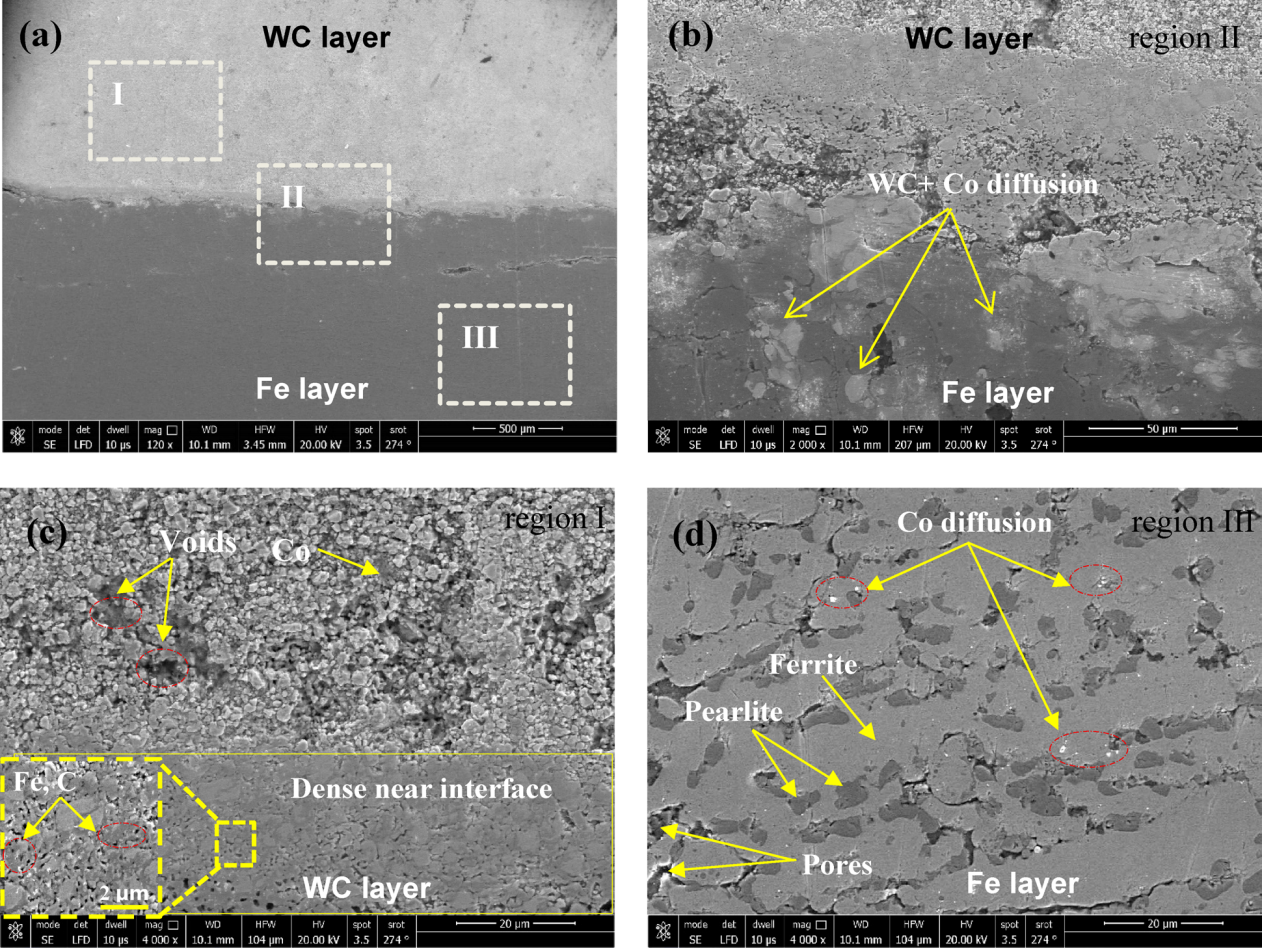
SEM microstructure of bimetallic composite Sp. 9 sintered at 1300 °C: **(a)** Overview of WC-Co/LCS interface; **(b)** Enlarged view of region II; **(c)** Enlarged view of region I; **(d)** Enlarged with mag. at 4000×view of region III.

In Fig. 9(a), SEM micrographs illustrate the LCS/WC–Co bilayer composite fabricated from fine powders (sub-25 μm) under 313 MPa compaction and sintered at 1300 °C. The polished cross-section reveals three distinct interface zones. The magnified image of region II (Fig. 9b) highlights a transition layer formed through atomic interdiffusion and solid-state reactions. XRD confirms the generation of Fe₃W₃C intermetallic compounds and a CoFe solid solution. The reaction zone appears as a thin dark band without visible cracks or voids, evidencing strong metallurgical bonding under the optimized sintering conditions. These findings demonstrate that enhanced diffusion and interfacial reactions, promoted by the sintering temperature, are responsible for the improved bonding strength of the joint.

SEM of region I (Fig. 9c) shows a typical cemented carbide microstructure, where the bright WC grains are surrounded by ductile Co binder, which consolidates the microstructure and impedes the crack propagation. Residual porosity near the free surface indicates incomplete densification at 1300 °C, requiring temperatures of ≥ 1350 °C for full sintering^52^. Nonetheless, the enhanced densification is observed just above the interface area due to interdiffusion of Fe and Co, which significantly enhances the local densification. Thereby reinforcing the cohesion of WC–Co composite. Region III (Fig. 9d) depicts the LCS substrate, presenting a dual-phase ferrite–pearlite structure. As shown, the bright ferritic regions alternate with darker pearlite, which composed of ferrite and cementite lamellae. Such a microstructure is highly sensitive to processing and heat treatment. The overall SEM analysis confirms the defect-free interfacial bonding, with Fe and Co diffusion driving the reaction-layer formation and ensuring the excellent structural integrity under the tested conditions (1300 °C, 313 MPa, sub-25 μm powders).

**Fig. 10:**
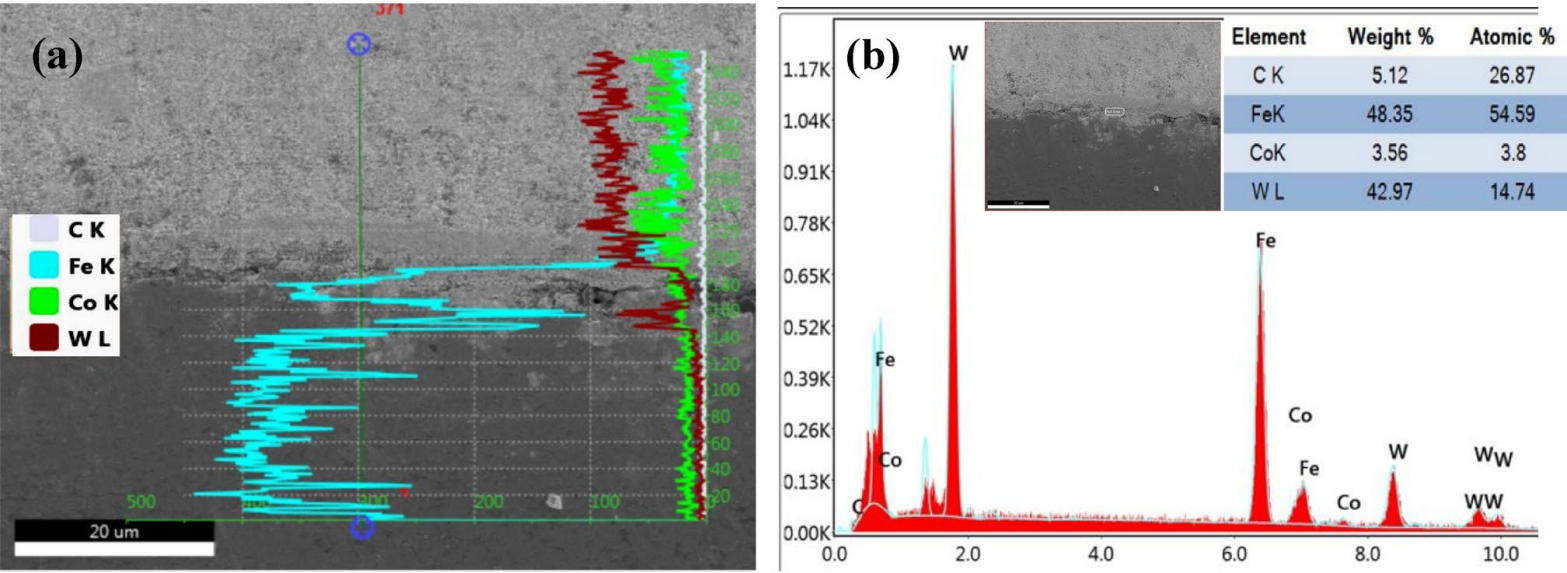
EDX of bimetallic composite Sp. 9 through interface region: **(a)** line scan; **(b)** EDX of full area.

The EDX line scan (Fig. 10a) reveals pronounced mutual diffusion, with W and Co diffuse downward into the steel substrate, while Fe migrates upward into the WC–Co layer. This interdiffusion, activated by the thermal energy of sintering, occurs as atoms overcome the lattice energy barriers and migrate via established diffusion mechanisms. Considering the atomic radii of W (137 pm), Fe (126 pm), Co (125 pm), and C (70 pm), the larger atoms (W, Fe, Co) primarily diffuse through a vacancy mechanism, whereas the smaller C atoms migrate interstitially. The full-area EDX spectrum (Fig. 10b) confirmed the presence of Fe (48.35 wt%), W (42.97 wt%), Co (3.56 wt%), and C (5.12 wt%) in the scanned region, reflecting a chemically mixed zone likely resulting from interfacial reactions during sintering. The presence of these elements in significant proportions suggests the formation of intermetallic compounds and complex carbides, in agreement with the XRD findings. Together, these results confirm the occurrence of strong elemental interdiffusion and the development of a diffusion based bonding mechanism at the interface, therby contributing to the mechanical integrity of the composite.

### Hardness test

**Fig. 11:**
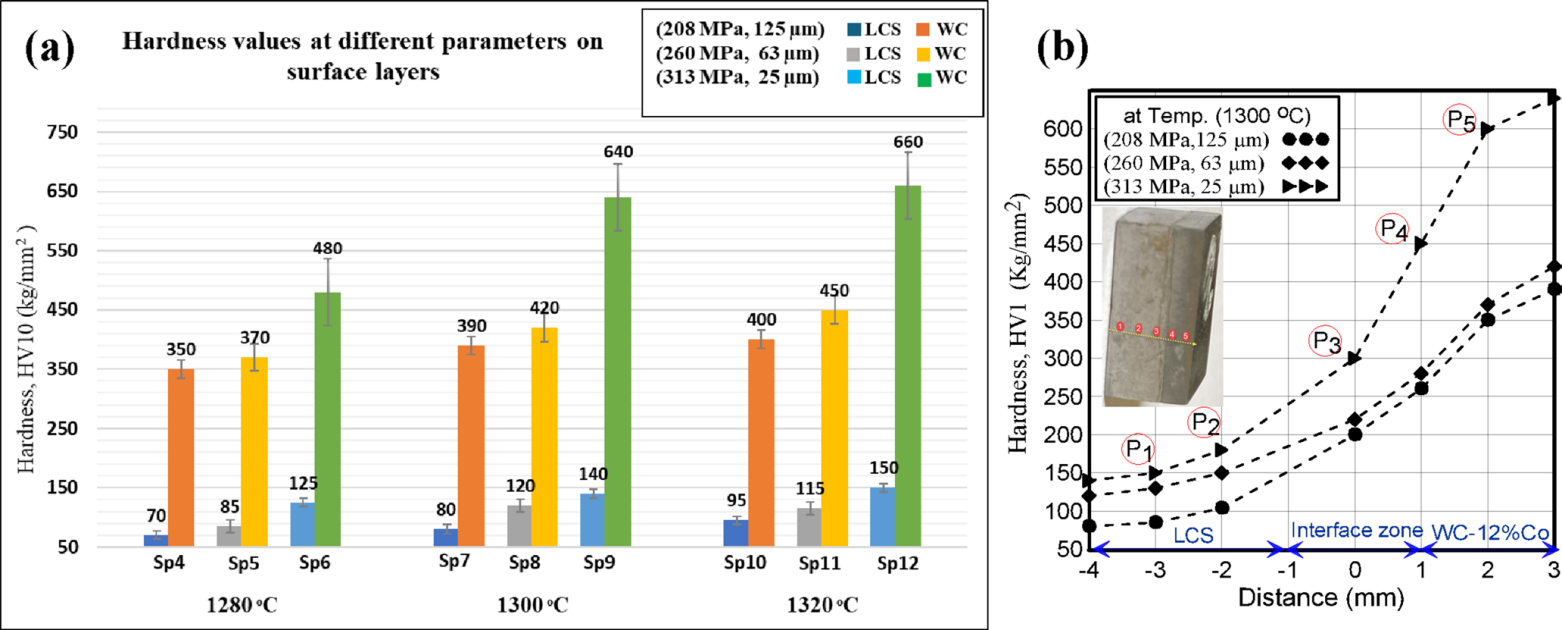
Average hardness values: **(a)** All bilayer specimens on the surface of layers (WC-12%Co) and LCS at processing parameters; **(b)** Hardness distribution along the cross-section of bilayer samples at 1300^°^C.

Figure 11(a) presents the hardness values of the outer surfaces of LCS substrate and WC-Co overlayer in the sintered bilayer composites. As seen, a gradual increase in hardness values with rising sintering temperature is evident in both layers. At lower temperatures, the inadequate densification of WC–Co layer results in porosity and voids with lowering the hardness values. Conversely, the higher sintering temperatures enhance densification, refine the microstructure, and strengthen the interfacial adhesion, while also promoting the favorable interlayer reactions^35^. Nonetheless, the sintering temperature must be precisely controlled to optimize bonding while preventing LCS layer melting or structural compromise. Hardness measurements revealed a strong dependence on particle size, with specimens fabricated from 25 μm particles at 313 MPa showing the highest values. This behavior follows the Hall–Petch relationship, where finer grains and improved densification enhance hardness^53^.

As shown in Fig. 11(b), the hardness distribution across the bilayer increases gradually from the LCS substrate to the WC–Co overlayer, confirming that the thin WC coating enhances the surface hardness of LCS. This gradient also reflects the toughness contrast, with the WC–Co side exhibiting higher hardness but greater brittleness, while the LCS side shows lower hardness consistent with its higher toughness.

Under optimized conditions (25 μm/313 MPa, 1300 °C), hardness reached 110 HV for pure LCS and 800 HV for WC–Co. With the WC overlay, the LCS surface hardness increased to 140 HV (27% rise) due to interlayer interactions, while the WC layer hardness decreased by 20% because of Fe diffusion and WC consumption, as confirmed by XRD (Fig. 8) showing Fe(W) and CoFe phases. These transformations reduce WC–Co hardness, consistent with the principle that coarse WC grains and higher cobalt content lower hardness but enhance toughness^54^.

### Diametrical compressive test

**Fig. 12:**
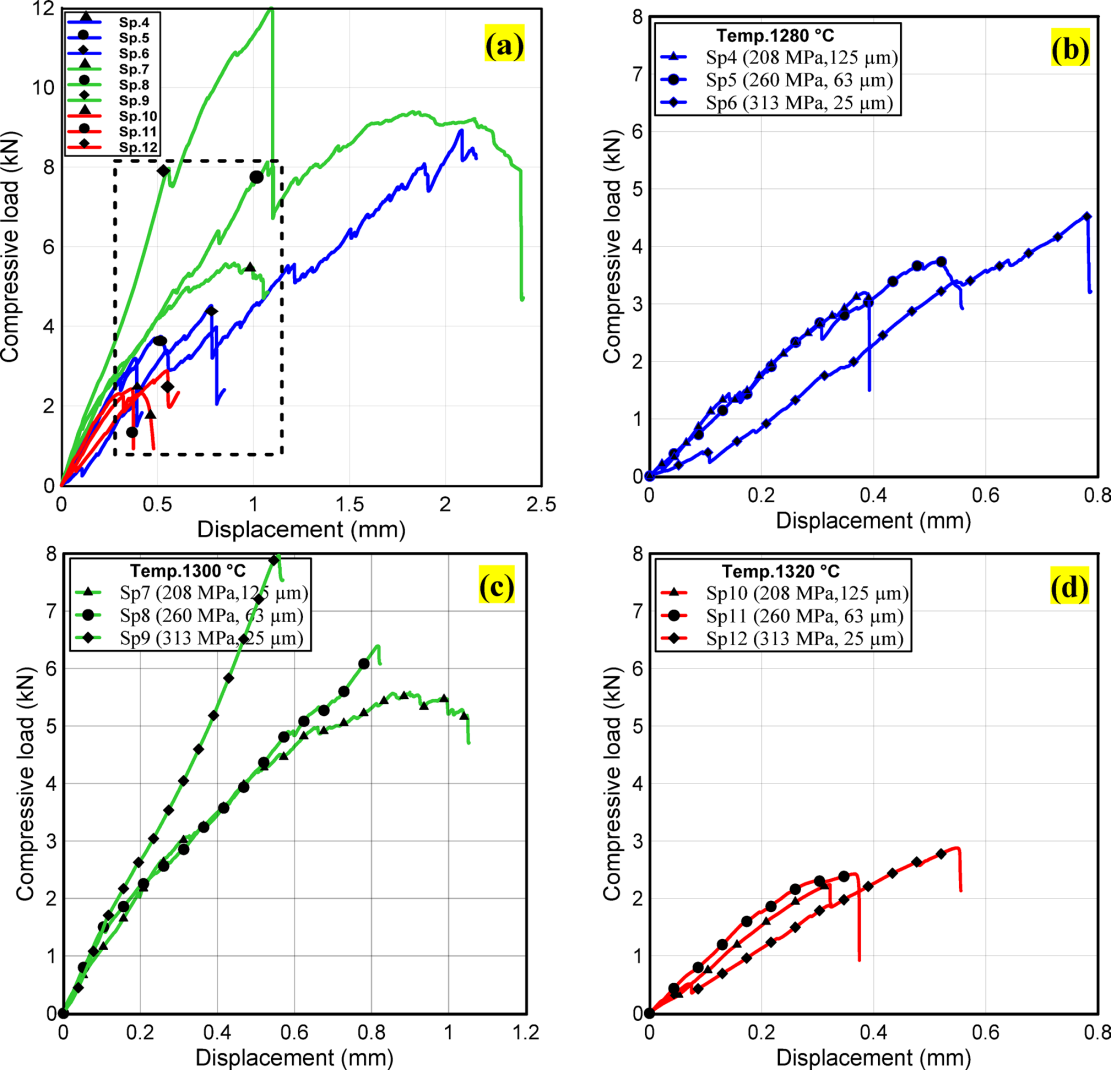
**(a)** Output load – displacement curve of diametrical compressive test for all samples, start of crack initiation at the bilayer interface at: **(b)** 1280 ᵒC, **(c)** 1300 ᵒC and **(d)**1320 ᵒC.

Figure 12 presents the load–displacement curves obtained from the diametrical compression test of the bilayer specimens. At the initial stage, a linear response is observed, corresponding to elastic deformation, until a distinct inflection point indicates the onset of interfacial cracking. This abrupt drop in load represents the failure of the interfacial bond and is utilized to evaluate bonding strength. Beyond this stage, the curve becomes nonlinear as cracks extend along the interface, ultimately resulting in delamination and tensile failure. The inset of Fig. 12(a) highlights the load–displacement curves up to the initiation of interfacial cracks between the two layers at different sintering temperatures, with detailed separation illustrated in Fig. 12(b-d). Two key findings emerge from these results. Firstly, specimens sintered at 1300 °C exhibited the highest compressive strength, followed by those at 1280 °C, while the lowest values were recorded at 1320 °C. This behavior is explained by improved interfacial bonding, as confirmed by optical and microstructural analyses, along with the highest densification in the 1300 °C samples. Secondly, compressive strength was observed to decrease with increasing particle size and lower compaction pressure. This reduction is linked to the presence of pores and voids that act as stress concentrators, serving as crack initiation sites. Furthermore, microstructural evaluation confirmed that larger particle sizes (125 μm and 63 μm) were associated with weaker interfacial adhesion, particularly at the bonding interface.

**Fig. 13:**
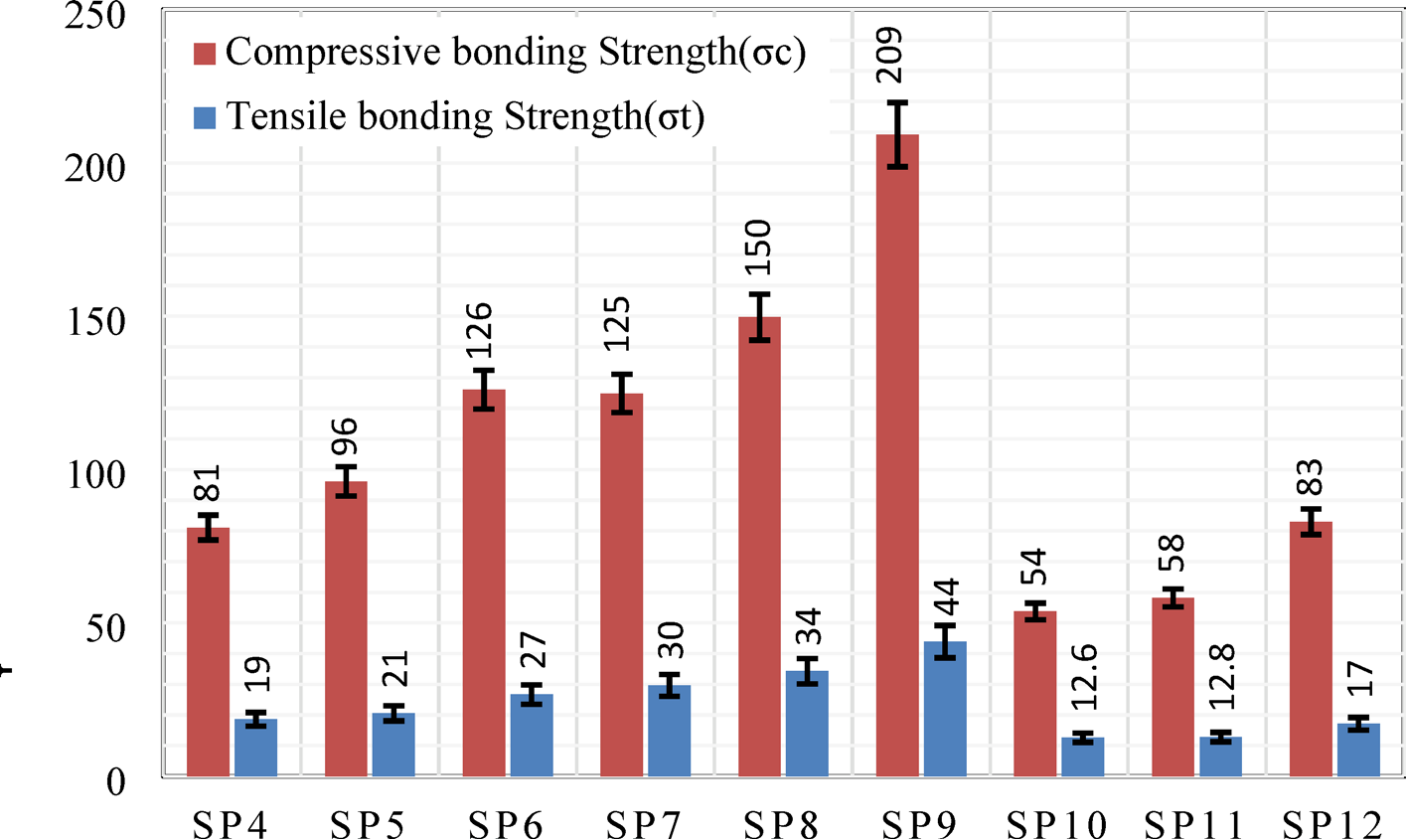
Interface bonding strength obtained from diametrical compression tests.

Figure 13 shows the compressive and tensile bonding strengths from the diametrical compression test, highlighting the bonding performance at the LCS/WC–Co interface. Specimens sintered at 1300 °C achieved the highest values due to strong interfacial bonds formed by interdiffusion and new reaction phases, while those at 1320 °C exhibited the lowest values because of partial melting, crack formation, and thermal mismatch induced stresses. In all cases, the compressive bonding strength exceeded the tensile bonding strength, owing to higher localized stresses under compression^45^.

After testing, all specimens showed a uniform failure pattern, with the WC overlayer detaching from the LCS substrate and fracturing into two halves, as shown in Fig. 4(c). This confirms the validity of the testing procedure and indicates that the tensile stresses specifically reflect the measuring interfacial bonding and cohesion strength at the LCS/WC–Co interface^41^. Compared with previous studies^41,55–57^, the present work identifies optimum processing at 25 μm/313 MPa/1300 °C, producing a dense bilayer with strong interfacial bonding. As shown in Table 4, the achieved bonding strengths are comparable to reported values on par with, or in some cases exceed, those previously reported despite differences in composition and conditions, while the slightly lower hardness can be attributed to the distinct microstructural features of our samples, indicating improved toughness.

**Table 3:** Manufacturing parameters of specimens using Tagushi(OAs) L_15_.

No**.**	Compaction pressure (MPa)	Particles size (LCS and WC) (μm)	Sintering temperature (◦C)
Sp1	208	125	
Sp2	260	63	1260
Sp3	313	25	
Sp4	208	125	
Sp5	260	63	1280
Sp6	313	25	
Sp7	208	125	
Sp8	260	63	1300
Sp9	313	25	
Sp10	208	125	
Sp11	260	63	1320
Sp12	313	25	
Sp13	208	125	
Sp14	260	63	1340
Sp15	313	25	

**Table 4:** Comparison of mechanical and microstructural properties of tungstenــsteel bilayer materials reported in previous studies.

Materials & Method	Processing conditions	Mechanical property	Microstructural Property	Refs.
W/Fe (FGM); By Resistance sintering under ultra-high.	pressure 9 GPa, 11 KW for 60 s.	Max hardness is **~ 725 HV** and **500 HV** for as-built and heat-treated, respectively.	Brittle Fe7W6 formed	^1^
WC/Fe bilayer with different C_gr_ addition to Fe part composition; By powder metallurgy.	sintering at 1280 °C for 1 h.	**HV**_**1**_ values of **735.7** & **151 kgf mm**^**−2**^ for WC & Fe layers; bonding tensile strength **55.75 MPa** (0.8wt.% C_gr_).	Fe_3_W_3_C (M_6_C) __ η phase	^2^
WC–Co/Ni composite (FGWC) and 410 stainless steel (410ss); By diffusion bonding in a vacuum furnace.	2 MPa from 850 to 1100 °C for a holding time of 20–120 min.	Max. tensile strength: WC–Co/410ss (**58 MPa**); FGWC/410ss (**195 MPa**) at 950 C for 80 min.	ـــــ	^3^
W/steel (EUROFER97) Cu foils interlayer; By FAST (electric field-assisted sintering) joining.	Sintered 980 °C; 2/5/9 min; pressure 42 MPa.	Shear strength = **48 ± 9 MPa** (2 min), **120 ± 31 MPa** (5 min), **81 ± 14 MPa** (9 min). HV_0.1_ values **450**; **100** and **390** for **W**; **Cu** and EUROFER97.	ـــــ	^4^
Recycled LCS/WC-Co bilayer composite via conventional powder metallurgy (PM).	Mesh size 125,63 and 25 μm under 208, 260, and 313 MPa sintering between 1260–1340 °C	Compressive and tensile interfacial bonding strength of **209** MPa and **44** MPa, with hardness of **150 ± 6 & 660 ± 70** HV for **LCS & WC–Co** bilayers.	Fe(W); CoFe, with Co₃W₃C; Fe₃W₃C intermetallic phases	Present study

## Conclusions

This work developed the recycled LCS/WC–12%Co bilayer composites by optimizing sintering temperature, particle size, and compaction pressure to improve hardness and interfacial bonding strength while modifying thermal mismatch. The major findings are summarized as follows:

1. The optimum processing parameters were identified at 1300 °C sintering temperature, 313 MPa compaction pressure, and 25 μm particle size, providing superior interfacial bonding strength and mechanical integrity.
2. Microstructural observations demonstrated poor adhesion at 1260 °C due to insufficient densification, and reduced bonding strength at 1340 °C caused by excessive sintering.
3. The density increased steadily up to 1300 °C and then declined at 1320 °C with maximum densification occurring at 313 MPa under 25 μm particle size.
4. Hardness measurements confirmed the improvements with decreasing particle size and increasing sintering temperature. Specifically, the LCS substrate hardness increased from 110 HV to 150 ± 6 HV through diffusion-driven phase formation, while the WC–Co layer reached 660 ± 70 HV, slightly below the theoretical maximum.
5. Specimens processed under optimal conditions (1300 °C, 313 MPa, 25 μm) achieved compressive and tensile bonding strengths of 209 MPa and 44 MPa, respectively. Conversely, specimens fabricated at 1320 °C, 208 MPa, and 125 μm exhibited minimal bonding strengths of 54 MPa and 12.6 MPa due to thermal degradation and coarse particle effects.

## Data Availability

All data generated or analyzed during this study are included in the present article. Derived data supporting the findings of this study are available from the corresponding author upon request.
